# Integrating omics datasets with the OmicsPLS package

**DOI:** 10.1186/s12859-018-2371-3

**Published:** 2018-10-11

**Authors:** Said el Bouhaddani, Hae-Won Uh, Geurt Jongbloed, Caroline Hayward, Lucija Klarić, Szymon M. Kiełbasa, Jeanine Houwing-Duistermaat

**Affiliations:** 1 Dept. of Biomedical Data Sciences, LUMC, Albinusdreef 2, Leiden, 2300 RC The Netherlands; 2 Department of Biostatistics and Research Support, UMC Utrecht, div. Julius Centre, Huispost Str. 6.131, Utrecht, 3508 GA The Netherlands; 3 Delft Institute of Applied Mathematics, EEMCS, TU Delft, Van Mourik Broekmanweg 6, Delft, 2628 XE The Netherlands; 4 Dept. of Statistics, University of Leeds, Leeds, LS2 9JT United Kingdom; 5 Genos Glycobiology Laboratory, Zagreb, 10000 Croatia; 6 MRC Human Genetics Unit, Institute of Genetics and Molecular Medicine, University of Edinburgh, Edinburgh, EH4 2XU Scotland; 7 Usher Institute of Population Health Sciences and Informatics, University of Edinburgh, Edinburgh, EH8 9DX Scotland

**Keywords:** Omics data integration, Joint principal components, Data-specific variation, R package, O2PLS

## Abstract

**Background:**

With the exponential growth in available biomedical data, there is a need for data integration methods that can extract information about relationships between the data sets. However, these data sets might have very different characteristics. For interpretable results, data-specific variation needs to be quantified. For this task, Two-way Orthogonal Partial Least Squares (O2PLS) has been proposed. To facilitate application and development of the methodology, free and open-source software is required. However, this is not the case with O2PLS.

**Results:**

We introduce **OmicsPLS**, an open-source implementation of the O2PLS method in R. It can handle both low- and high-dimensional datasets efficiently. Generic methods for inspecting and visualizing results are implemented. Both a standard and faster alternative cross-validation methods are available to determine the number of components. A simulation study shows good performance of OmicsPLS compared to alternatives, in terms of accuracy and CPU runtime. We demonstrate OmicsPLS by integrating genetic and glycomic data.

**Conclusions:**

We propose the OmicsPLS R package: a free and open-source implementation of O2PLS for statistical data integration. OmicsPLS is available at https://cran.r-project.org/package=OmicsPLS and can be installed in R via install.packages(“OmicsPLS”).

**Electronic supplementary material:**

The online version of this article (10.1186/s12859-018-2371-3) contains supplementary material, which is available to authorized users.

## Background

With rapid advances in technology, several levels of biological variation can be measured. Consequently, multiple omics data sets are available on the same set of subjects. For a better understanding of the underlying biological systems, these data should be analyzed simultaneously [1].

Several data integration methods have been developed that estimate joint parts while ignoring some of the data-specific characteristics. For example, Partial Least Squares (PLS) [2] maximizes the covariance to calculate joint principal components. Canonical Correlation Analysis (CCA) [3] considers correlation rather than covariance. Several other methods perform analysis on a concatenated version of the data sets, such as Simultaneous Component Analysis (SCA) [4]. For many data integration methods, open source software packages are available [5]. In particular, the mixOmics R package implements several variants of PCA, PLS and CCA [6].

Omics data sets might be heterogeneous in that they typically differ in data-specific characteristics, such as size, scale, distribution and experimental error. This hampers the estimation of joint parts between these data. For correct interpretation of data integration results, data-specific variation should be modeled [7]. This variation captures information specific to each data set. Furthermore, it can distort interpretation of the estimated joint part [8]. Therefore, we consider approaches that estimate both joint and specific components. Such approaches include Two-Way Orthogonal PLS (O2PLS) [8], JIVE [9] and DISCO-SCA [10]. O2PLS considers two data sets and decomposes both in a joint, specific and residual part. The joint parts are calculated by maximizing the covariation between the two data sets, while correcting for data-specific variation. JIVE uses iterative PCA on the concatenation of multiple datasets to alternately find joint and data-specific parts. DISCO-SCA performs SCA and rotates the solution to obtain joint and specific components for each data set.

In the JIVE and DISCO-SCA approach, the joint and specific components are constrained to be orthogonal to each other. Moreover, they assume that the data sets share exactly the same joint latent variables. O2PLS only imposes orthogonality of the components within each part and assumes correlated joint latent variables for each data set. Therefore, we expect a better performance of O2PLS in complex situations.

O2PLS is implemented within the software package SIMCA [11], which is closed-source and commercial. Unavailability of source code hampers developing and extending the methodology. No free and open source alternative implementing O2PLS is available to the best of our knowledge. Therefore, we propose OmicsPLS, a free and open-source R software package to decompose two datasets into joint and specific parts. With regard to the other methods, DISCO-SCA [12] is available only from the commercial computing environment MATLAB, whereas JIVE is freely available in the r.jive package [13]. Therefore, we compare OmicsPLS to r.jive.

Our aim is to provide easy access to both the method and visualization tools and to facilitate the development of more advanced methodology. The rest of the article is organized as follows. First, we discuss the implementation of OmicsPLS in detail. Second, the OmicsPLS package is illustrated using genetic and glycan data from a Croatian population cohort. We also apply JIVE to these data. Motivated by the data analysis, we conduct a simulation study to compare OmicsPLS to r.jive in terms of estimation accuracy, execution time and robustness against the presence of data-specific characteristics. Finally, we discuss future extensions of OmicsPLS.

## Implementation

### O2PLS model

Let the observed data be collected in a matrix *X*=[*x*_1_,…,*x*_*p*_] (*N*×*p*) and a matrix *Y*=[*y*_1_,…,*y*_*q*_] (*N*×*q*). Here, *N* denotes the number of subjects, and *p* and *q* denote the number of variables in *X* and *Y*, respectively. The O2PLS method decomposes *X* and *Y* in two joint, specific and residual parts. The dimension of the joint part is given by *n*, the dimension of each specific part is given by *n*_*X*_ and *n*_*Y*_, respectively. The joint parts consist of matrices *T*, *U* (both *N*×*n*), *W* (*p*×*n*) and *C* (*q*×*n*). The matrices *T* and *U* are referred to as joint scores or joint latent components, and the matrices *W* and *C* are referred to as joint loadings or joint principal components. These joint parts represent the statistical overlap between *X* and *Y*. The specific parts consist of matrices *T*_*Y*⊥_ (*N*×*n*_*X*_), *U*_*X*⊥_ (*N*×*n*_*Y*_), *P*_*Y*⊥_ (*p*×*n*_*X*_) and *P*_*X*⊥_ (*q*×*n*_*Y*_). These matrices are referred to as specific scores and loadings, respectively. The residual parts are denoted by *E* (*N*×*p*) and *F* (*N*×*q*). Then, the O2PLS decomposition is

1 $$ \begin{aligned} {X} & = TW^{\top} + T_{{Y} \bot}P_{{Y} \bot}^{\top} + {E}, \\ {\underset{{Data}}{\underbrace{{Y}}}} & = {\underset{{Joint}}{\underbrace{{UC^{\top}}}}} + {\underset{{Specific}}{\underbrace{{U_{{X} \bot}P_{{X} \bot}^{\top}}}}} + {\underset{{Residuals}}{\underbrace{{F}}}}. \end{aligned}   $$

Each row of *X* and *Y* contains measurements on *the same* subject. Throughout the paper, it is assumed that the columns of *X* and *Y* are centered around zero. The relationship between *T* and *U* is given by the linear model *U*=*TB*_*T*_+*H* or *T*=*UB*_*U*_+*H*^′^. Here, *B*_*T*_ and *B*_*U*_ are square matrices of size *n*, representing regression coefficients for the two models. The particular choice of the model does not affect the estimates, as the O2PLS algorithm is symmetric in *X* and *Y*.

Note that, in PLS, only a joint and a residual part is considered for each data set. Any data-specific variation is absorbed by these two parts. This makes interpretation of PLS results more difficult, as the estimated loadings may be biased and the correlation between the joint scores typically seem weaker. O2PLS restricts the joint loadings *W* and *C* and the specific scores *T*_*Y*⊥_ and *U*_*X*⊥_ to have orthonormal columns. JIVE and DISCO-SCA additionally restrict the columns of the matrices [ *WP*_*Y*⊥_] and [ *CP*_*X*⊥_] to be orthonormal. Furthermore, both methods assume that *U*=*T*, while O2PLS only assumes a linear relation between *U* and *T*.

The O2PLS algorithm for estimating the O2PLS components is provided in [8]. Briefly, singular vectors of the covariance matrix *X*^⊤^*Y* are calculated. From these vectors, loadings and scores containing both joint and specific variation are estimated. Then, specific variation is estimated using SVD and subtracted from the data. Finally, using the corrected data, the joint parts are re-estimated.

**Interpretation** Within each part, the components have a similar interpretation as PCA. In particular, the loading value *w*_*jk*_ indicates the importance of the *variable* *x*_*j*_ for component *k*. If *w*_*jk*_ and $\phantom {\dot {i}\!}w_{j^{\prime }k}$ have the same sign, the corresponding variables *x*_*j*_ and $\phantom {\dot {i}\!}x_{j^{\prime }}$ are positively correlated within component *k*. The same interpretation holds for the other parts. The scores can be used to define similarity between *subjects* within each component: for example, if $\phantom {\dot {i}\!}t_{ik} \approx t_{i^{\prime }k}$, then subjects *i* and *i*^′^ are similar in component *k*. Between the joint parts, in the *k*’th joint component, the loading values *w*_*jk*_ and $\phantom {\dot {i}\!}c_{j^{\prime }k}$ indicate correlation between *x*_*j*_ and $\phantom {\dot {i}\!}y_{j^{\prime }}$. High positive or negative loading values indicate high positive or negative correlation in this component between these variables, respectively. As a consequence, the joint loading values *w*_*k*_ and *c*_*k*_ can be sorted to prioritize variables in *X* and *Y* based on high covariation.

### Implementation

The functions in OmicsPLS can be organized as follows

- Cross-validating: Functions to determine the number of O2PLS components.
- Fitting: Functions to fit the O2PLS model.
- Summarizing & visualizing: Functions to summarize and visualize the results.

**Cross-validating.** Cross-validation is a well-known technique to choose tuning parameters of a model, while limiting the risk to overfit. All samples are partitioned in *k* blocks (denoted as folds), and the model is fitted on *k*−1 folds. The left out fold is used to evaluate the model fit. For O2PLS, an approach to determine the number of components is to maximize the prediction error over a three-dimensional grid of possible integers and select the triple (*n*,*n*_*X*_,*n*_*Y*_) that minimizes this error. As O2PLS is symmetric in *X* and *Y*, the sum of the two prediction errors $||Y - \hat {Y}||^{2}+||X - \hat {X}||^{2}$ is taken as error measure. Here, $||A||^{2} := \sum _{ij} a_{ij}^{2}$. This approach is implemented in the crossval_o2m function:

crossval_o2m(X, Y, a, ax, ay, nr_folds)

Here, a, ax and ay are vectors of integers to consider for the number of components *n*, *n*_*X*_ and *n*_*Y*_. The vector a must have positive elements, while both ax and ay may contain zeros. The number of folds is specified by nr_folds and should be between two and *N*. The crossval_o2m function returns a three-dimensional array with the prediction errors.

Cross-validation over a three-dimensional grid can be computationally intensive, especially with many grid points. For this reason, we have proposed an alternative cross-validation procedure [14]. The rationale behind this approach lies in the interpretation of the specific parts: specific variation in the data will affect the joint scores, thereby reducing the covariance between *T* and *U*. Correcting for specific variation will increase this covariance. On the other hand, overcorrecting will again reduce the covariance between the joint scores. Candidates for *n*_*X*_ and *n*_*Y*_, given *n*, are those integers for which the covariance of the joint scores are maximized. This approach is called by:

crossval_o2m_adjR2(X, Y, a, ax, ay, nr_folds)

It performs the cross-validation over a one-dimensional grid a, while maximizing the covariance between the joint scores *T* and *U* over a two-dimensional grid given by ax and ay. The last maximization does not involve cross-validation. Consequently, the looping over nr_folds folds is omitted in two dimensions. This can drastically reduce computation time, while often yielding similar minimizers to those obtained with the full cross-validation approach. The output is a matrix containing the prediction errors and the number of components (*n*,*n*_*X*_,*n*_*Y*_).

Note that these two cross-validation strategies can be combined: The alternative cross-validation is used to find candidate minimizers of the prediction error. Based on these minimizers, a three-dimensional grid is constructed on which the full cross-validation is performed. Both cross-validation implementations support parallel computation.

**Fitting** In its simplest form, the function call for fitting the O2PLS model is o2m(X, Y, n, nx, ny) The input parameters are the two data matrices *X* and *Y*, and the number of components in the joint, *X*-specific and *Y*-specific part. The output is a list containing scores and loadings in the notation of [8], as well as proportions of explained variance and residual matrices; these proportions are defined below. The user can choose a ‘stripped’ output, by adding stripped=TRUE as an argument, to discard the residual matrices (and reduce memory usage).

By default, a Singular Value Decomposition (SVD) of the covariance matrix between *X* and *Y* is used to calculate joint and specific components. If both *X* and *Y* are high-dimensional, the covariance matrix *X*^⊤^*Y* will use a high amount of memory. Therefore, an alternative algorithm is implemented in the OmicsPLS package, named NIPALS [2]. The NIPALS algorithm is an iterative algorithm that avoids construction and storage of the covariance matrix. Moreover, the NIPALS-based joint components are numerically equal to the SVD-based PLS components (up to sign) if the number of NIPALS iterations is large enough. In the case that *p* or *q* is not too large, the NIPALS approach can be slower than the SVD approach. Therefore, a check on data dimensionality is performed to determine the proper approach. The threshold is by default at *p*=*q*=3000 and can be adjusted.

**Summarizing & visualizing** A summary of the modeled variation is given by summary(object). Here, object contains the O2PLS fit as produced by the o2m call. The output includes proportions of:

- variation in *X* and *Y* explained by the joint, specific and residual parts, e.g., ||*TW*^⊤^||/||*X*||.
- variation in *U* and *T* that is predictable by *T* resp. *U*, e.g., ||*TB*_*T*_||^2^/||*U*||^2^.

Note that the proportion of predictable variation in *Y* by *X* is then ||*TB*_*T*_||^2^/||*U*||^2^×||*UC*^⊤^||^2^/||*Y*||^2^=||*TB*_*T*_||^2^/||*Y*||^2^.

The OmicsPLS package provides a flexible framework to plot loadings in each component. As this framework is built on the ggplot2 package, several plotting layers can be added to enhance visualization and aid interpretation of the results. The command for constructing a plot is

plot(x, loading_name).

Here x is the O2PLS fit and the only required object. The parameter loading_name represents which of the four parts (X-joint, Y-joint, X-specific or Y-specific) should be plotted. The plot command calls geom_text from the ggplot2 package. Its documentation contains information about editing, for example, text color, transparency and size. These attributes can be changed within the OmicsPLS plot function.

**Fig. 1:**
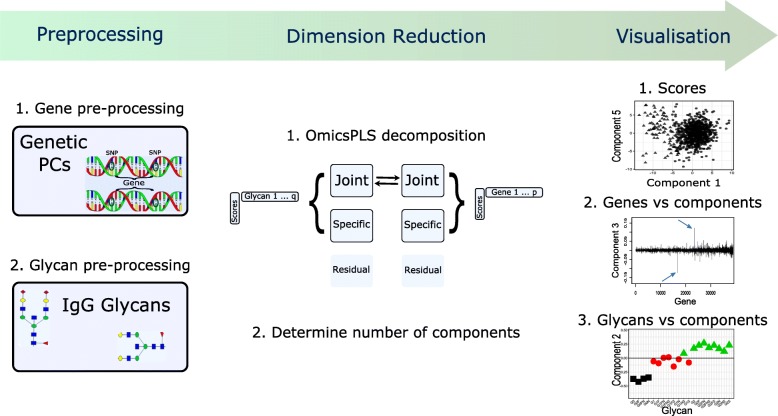
Workflow of the OmicsPLS package. Firstly, each data set is pre-processed. Secondly, O2PLS is used to decompose each data set in joint, specific and residual parts. Finally, the output is visualized and interpreted

**Workflow and tutorial** A workflow for OmicsPLS analysis is provided in Fig. 1. The steps in the workflow are based on the genetic and glycomic data analysis showed the next section. Furthermore, a tutorial is available as an online supplement, illustrating OmicsPLS with freely available transcriptomic and metabolomic data (Additional file 1).

## Results

### Analysis of genetic and glycomic data

We consider *p*=333858 genotyped Single Nucleotide Polymorphisms (SNPs) and *q*=20 quantified IgG1 glycan (glycopeptide) abundances, measured with nano-LC-ESI-MS, for *N*=885 participants in the CROATIA_Korcula cohort [15]. Both data sets contain highly correlated measurements and are heterogeneous (as they differ in scale, distribution and measurement error).

Our aim is to investigate how IgG1 glycans relate to genetic variation by determining the statistical overlap between IgG1 glycan data and genetic data, as in Eq. (1). To this end, we use the OmicsPLS package to obtain estimates of the amount of joint variation and estimate the contribution of the genetic and IgG1 glycan measurements to this joint variation.

The SNPs were summarized by taking, for each gene (in the UCSC hg18 database), all SNPs within 50 kilobases from that gene and applying Principal Components Analysis. For each gene, the set of corresponding SNPs were replaced by as many principal components as needed to explain at least 80% of this set of SNPs. This provided a new data set with one or several variables, which we denote as Genetic PCs, per gene. This ‘Genetic PCs’ data set contains 37819 variables and is referred to as *X*. The glycan measurements were log-transformed, batch-corrected [16] and quantile-normalized [17]. The resulting data matrix is referred to as *Y*.

**Fig. 2:**
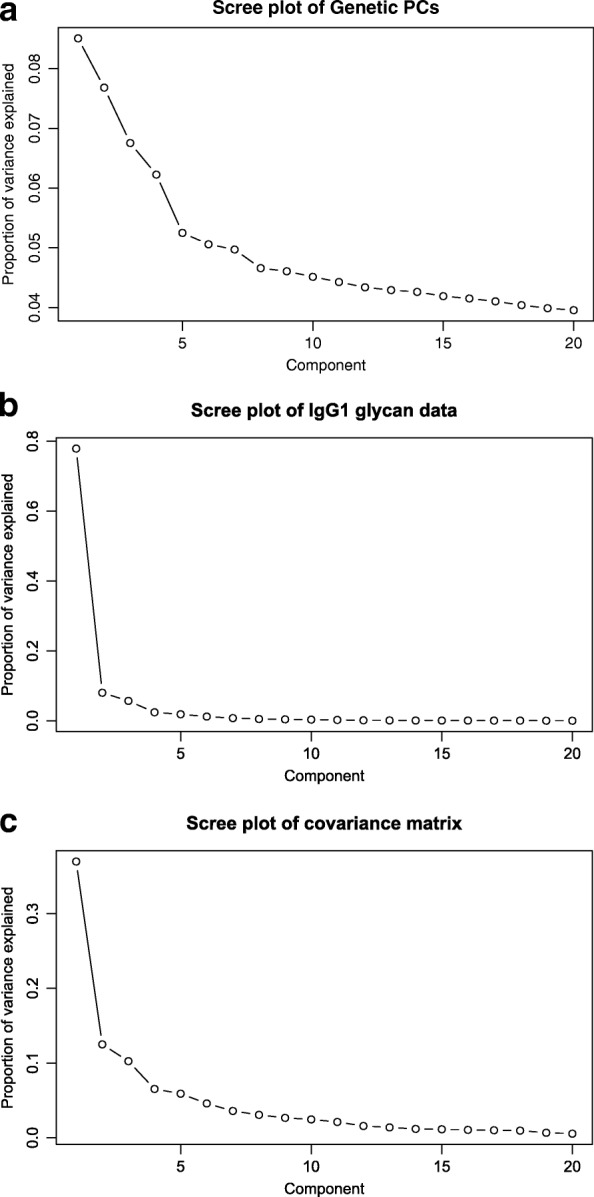
Eigenvalues of the covariance matrices of the genetic and glycan data. The relative contribution of each eigenvalue towards the sum of all eigenvalues is shown for the Genetic PCs (panel **a**) and IgG1 glycan data (panel **b**), and their covariance (panel **c**), respectively

Scree plots of *XX*^⊤^, *Y*^⊤^*Y* and *X*^⊤^*Y* are shown in Fig. 2. By identifying an elbow in these scree plots, the number of joint and specific components are determined. Based on the plots, 5 joint and 5 genetic-specific components were retained. Note that no glycan-specific parts were detected. The O2PLS fit took around 5 s.

**Fig. 3:**
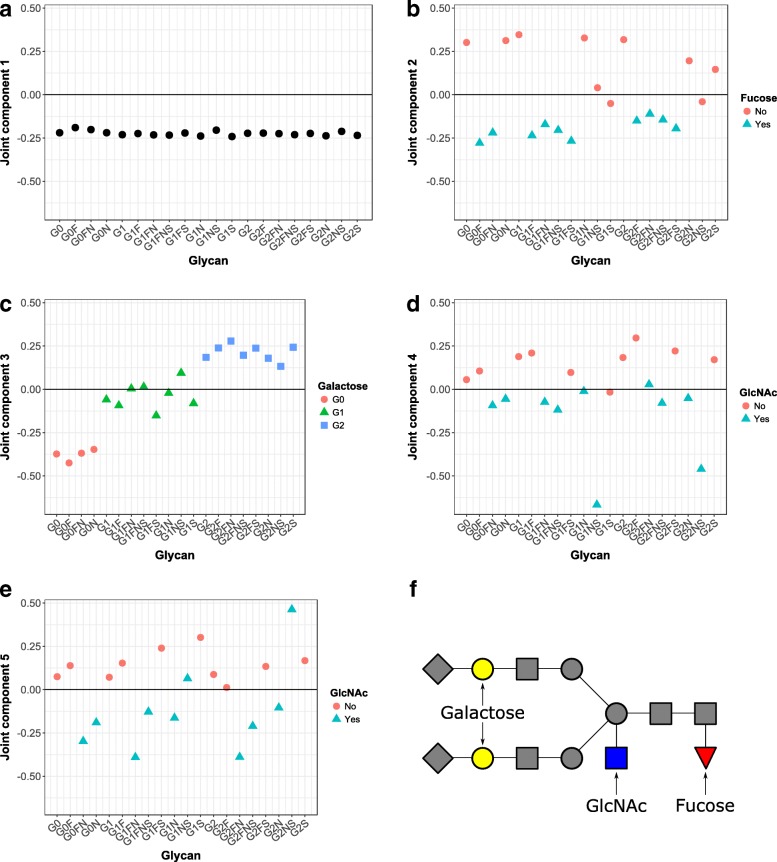
Genetic-Glycan joint principal components obtained with the OmicsPLS R-package. Loading values of each IgG1 glycan variable are depicted per component (panel **a**-**e**). The colors and shapes represent the biological grouping of the glycans. In the last row and column, a graphical representation of the structure of a particular glycan is shown (panel **f**)

Regarding the five IgG1 glycan joint components, they account for 96% of the total IgG1 glycan variation. The amount of IgG1 variation that can be predicted with the Genetic PCs is 70%. The loading values of each IgG1 glycan variable are depicted in Fig. 3. The first joint component is proportional to the ‘average’ IgG1 glycan, as all glycans get approximately the same loading value. The second joint component distinguishes fucosylated (negative loading values) and non-fucosylated (mostly positive loading values) IgG1 glycans. This component is referred to as the ‘fucosylation’ component. The third joint component involves especially non-galactosylated (negative loading values) and di-galactosylated (positive loading values) IgG1 glycans, while mono-galactosylated glycans have estimated loading values around zero. This component is referred to as the ‘galactosylation’ component. In the fourth joint component, G1NS and G2NS glycans have high loading values. The fifth joint component distinguishes, apart from G1NS and G2NS, glycans for which bisecting GlcNAc is present (negative loading values) or absent (positive loading values).

Regarding the five joint components in the Genetic PCs data set, they account for 0.8% of the total variation. For the specific parts, this percentage is 1.9%. The top five genes in each Genetic PCs joint component are shown in Table 1. In the first Genetic PCs joint component, the gene with the highest loading value is *DNAJC10*. The corresponding protein is involved in recognizing and degrading misfolded glycoproteins. This first joint component corresponds to the ‘average’ glycan pattern in the first glycan joint component. The top gene in the second joint component, which corresponds to the ‘fucosylation’ component, is *FUT8*. It encodes a fucosyltransferase enzyme that catalyzes the transfer of fucose to a glycopeptide. In the third joint component, which corresponds to the ‘galactosylation’ component, the gene *AKAP9* has second highest loading value. It encodes an A-kinase anchor protein, which is involved in maintaining the integrity of the Golgi apparatus. Note that in the Golgi apparatus, glycosylation (in particular galactosylation) takes place. In the fourth and fifth component, no directly relevant genes were found. More research is needed to further elucidate these relationships.

**Table 1:** Top 5 genes and loading values of the Genetic-Glycan joint principal components

Component 1: ‘average’ glycan		Component 2: ‘fucosylation’		Component 3: ‘galactosylation’	
Gene symbol	Loading value	Gene symbol	Loading value	Gene symbol	Loading value
DNAJC10	-0.0929	FUT8	-0.0844	MTO1	0.0875
ARID3B	-0.0880	LGALS8	-0.0781	AKAP9	-0.0627
ZNF502	0.0756	LDB3	0.0766	MRPL33	-0.0622
TBC1D13	0.0611	ARID3B	-0.0701	MYLPF	0.0562
ZC2HC1C	0.0601	LCE2D	-0.0677	POLR2F	0.0554

The results are displayed per component. Only the first three components are shown

For comparison purposes, r.jive was also applied to the data. However, the algorithm did not converge after 500 iterations (and 3000 s). We will investigate possible reasons in the simulation study.

### Simulation study

A simulation study is conducted to compare r.jive and OmicsPLS in terms of accuracy and speed. To gain insight into the robustness of r.jive, possible reasons for the lack of convergence of r.jive are investigated. The simulated data follow a model that satisfies the assumptions of both O2PLS and JIVE:

2 $$ \begin{aligned} X & = T W^{\top} + T_{{Y} \bot} P_{Y\perp}^{\top} + E, \\ Y & = U C^{\top} + U_{{X} \bot} P_{X\perp}^{\top} + F, \end{aligned}  $$

where *U*=*T*. Note that in the O2PLS formulation, *B*_*T*_=*I*_*r*_ and *H*=0. In the first scenario, we take *N*=500, *p*=*q*=100, *n*=2, *n*_*X*_=3 and *n*_*Y*_=1. In the second scenario, we consider *p*=*q*=10^4^. Elements of *W*, *C*, *P*_*Y*⊥_ and *P*_*X*⊥_ are drawn independently from a standard normal distribution. The JIVE constraints are applied by orthogonalizing each column in both joint and specific parts with respect to each other. Elements of *T*, *T*_*Y*⊥_ and *U*_*X*⊥_ are drawn independently from a standard normal distribution. Noise, represented by *E* and *F*, is added to *X* and *Y* to account for about 10% of the total variation. For both r.jive and OmicsPLS, loading matrices are extracted. To evaluate estimation accuracy, the absolute value of the inner product between corresponding columns are calculated. Here, higher values represent lower estimation errors. For each scenario, we generated 1000 replicates.

To investigate the lack of convergence of r.jive in the data analysis, two additional scenarios are considered. In the first additional scenario, elements in *U* have a standard deviation of 10, i.e., *U*=10*T*. In the second additional scenario, elements in the specific parts will be normally distributed with a standard deviation of 10. The dimensions and sample size are taken as above. Note that both scenarios represent an ‘imbalance’ in the amount of variation per part. Here, r.jive is considered converged if it needs less than 500 steps. In these additional scenarios, we generated 100 replicates.

In Table 2, median inner product values, together with Median Absolute Deviations (MAD) are shown for *p*=*q*=100. It can be seen that for balanced scenario settings, OmicsPLS performs as good as r.jive in terms of median inner product. The results for *p*=*q*=10^4^ were very similar to these results (not shown).

**Table 2:** Simulation results for OmicsPLS and r.jive: inner products

	OmicsPLS	r.jive
X joint	0.88 (0.09)	0.88 (0.09)
X specific	0.79 (0.08)	0.78 (0.09)
Y joint	0.85 (0.08)	0.85 (0.08)
Y specific	0.93 (0.013)	0.92 (0.014)

These results are for *p*=*q*=100. One thousand replicates were generated. Median (MAD) values of (the absolute value of) inner products between true and estimated loading vectors for O2PLS and JIVE. Higher values indicate better agreement with true loadings. The results are very similar for high-dimensional data (*p*=*q*=10^4^)

In Table 3, elapsed time and convergence ratios are shown. OmicsPLS runs about 3500 times faster in the first scenario (*p*=*q*=100) and 7 times faster in the second (*p*=*q*=10^4^) scenario. In both additional scenarios in which there is an imbalance in the amount of variation between the joint and specific parts, r.jive failed to converge in the majority of runs. In case *U*=10*T*, r.jive did not converge in more than 90% of the runs. In case the specific parts contain more variation, r.jive failed to converge in 74 and 63 out of 100 runs, for *p*=*q*=100 and *p*=*q*=10^4^, respectively.

**Table 3:** Performance comparison of OmicsPLS and r.jive w.r.t. median (MAD) total elapsed time in seconds across 1000 replicates, and convergence across 100 runs

	CPU time (sec)		Convergence (%)	
Dimensions	OmicsPLS	r.jive	OmicsPLS	r.jive
Low (*p*=*q*=100)	0.04 (0.007)	14 (2.8)	100	9
High (*p*=*q*=10^4^)	18 (4.1)	132 (16)	100	8

For the convergence, the heterogeneity scenario *U*=10*T* was used

R code for the data analysis and simulation study is available as an online supplement (Additional file 2).

## Discussion

In this article, we introduced the OmicsPLS package for integration of two (omics) data sets. We evaluated its performance with a simulation study and demonstrated it using genetic and IgG1 glycomic data. Regarding the data analysis, the proportion of joint variation in the Genetic PCs data set was 0.8%. This proportion is expected to be small since it is not likely that a large fraction of genetic variation (in particular SNPs) is related to IgG1 glycosylation. In the joint components, several genes were found that might play a role in the genetic regulation of IgG1 glycosylation. Some of these genes are known to be directly involved (e.g., *FUT8*), while others (*DNAJC10* and *AKAP9*) are localized to cell compartments where the majority of glycosylation takes place (the endoplasmic reticulum and Golgi Apparatus). However, much is still unknown about the genetic regulation of (IgG) glycosylation.

Additionally, we considered JIVE for this type of data, but without success: the algorithm did not converge. A potential cause for this lack of convergence is the different data-specific characteristics of the two data sets. In particular, the dimensionality and amount of variation differ. Therefore, the JIVE assumption *U*=*T* might not be reasonable. This is confirmed by our simulation: the r.jive algorithm is not robust against an ‘imbalance’ in the amount of variation between the two joint parts, or between the joint and specific parts. In particular, when *U*=10*T*, r.jive did not converge in more than 90% of the replicates. This suggests that r.jive might be inappropriate for analyzing heterogeneous data sets (in which data-specific characteristics differ across data sets). Note that in DISCO-SCA the same assumption (*U*=*T*) is made, therefore we expect a suboptimal performance of this method as well when analyzing heterogeneous data.

As part of a future update of the OmicsPLS software package, we intend to deal with missing data. To impute missing values and simultaneously estimate O2PLS components, the OmicsPLS algorithm can be extended [2]. The imputation step can also be performed prior to analysis. For multiple omics data, Ensemble Regression Imputation [18] and Multiple Factor Analysis imputation [19] have been proposed. Note that, as with all imputation methods, uncertainty due to missing data should be assessed and presented to the user. A probabilistic framework for O2PLS would facilitate imputation and simultaneously addresses additional uncertainty due to missing data.

An important extension of OmicsPLS involves obtaining standard errors for the estimates. To this end, bootstrap approaches, similar to those found in PLS literature, can be applied [20]. A drawback of using resampling methods is the computational burden, especially with high-dimensional data sets. To avoid such procedures, a probabilistic framework for O2PLS can be used to directly calculate asymptotic standard errors.

Interpretability of the OmicsPLS output can be increased by extending the algorithm to produce sparse estimates. This extension can be implemented by considering Sparse PLS [21] or by considering a probabilistic framework for O2PLS and obtaining penalized maximum likelihood estimates.

We are currently investigating the possibilities of Probabilistic O2PLS for data integration, which facilitates multiple imputation and statistical inference, such as calculation of asymptotic standard errors. By penalizing the likelihood, sparse estimates can be obtained.

As OmicsPLS is open-source, it is straightforward to extend the current implementation to handle more complex situations. For example, in the GitHub repository, several ‘branches’ can be initialized in which new functionalities can be developed.

## Conclusion

We propose OmicsPLS, an open-source and freely available R package for robust integration of heterogeneous data with O2PLS. It includes functions to determine the number of components, fit, and inspect results. For high-dimensional data, a memory-efficient implementation is used.

## Availability and requirements

- Project name: OmicsPLS
- Project home page: https://github.com/selbouhaddani/OmicsPLS
- Operating systems: Linux, Mac OS, Windows
- Programming language: R
- License: GPL-3
- Any restrictions to use by non-academics: none.

## Additional files

Additional file 1 A tutorial on using OmicsPLS. This pdf contains a case study illustrating the OmicsPLS package using freely available transcriptomics and metabolomics measurements from a Finnish population cohort. We discuss input and output of the main functions, interpret the analysis results and show how to generate publication-ready figures. (PDF 1172 kb)

Additional file 2 R code used in data analysis and simulation. This pdf contains the R code used to obtain results for the data analysis and simulation study. (R 6 kb)

## Abbreviations

Genetic PCs: Genetic principal components
JIVE: Joint and individual variances explained
O2PLS: Two-way orthogonal partial least squares
