# Supplementary material for: Integrating omics datasets with the OmicsPLS package
Source: BMC Bioinformatics. 2018 Oct 11;19:371. doi: 10.1186/s12859-018-2371-3 (PMC6182835; doi:10.1186/s12859-018-2371-3)
Supplement: Supplementary file 1 — A tutorial on using OmicsPLS. This pdf contains a case study illustrating the OmicsPLS package using freely available transcriptomics and metabolomics measurements from a Finnish population cohort. We discuss input and output of the main functions, interpret the analysis results and show how to generate publication-ready figures. (PDF 1172 kb) [file 12859_2018_2371_MOESM1_ESM.pdf]

The OmicsPLS R Package

Said el Bouhaddani

2018-02-08

The OmicsPLS R package

Welcome to the vignette of the O2PLS package for analyzing two Omics datasets!

Here you can find examples and explanation of the input options and output objects. After installing, help is found with the `?` operator. Try to type `?OmicsPLS` for an overview of the package and `?o2m` for description of the main fitting function.

Installing and loading

The easiest way to install the OmicsPLS package is to run `install.packages("OmicsPLS")`. If the command did not work, check if there is a package missing. It imports the **ggplot2** and **parallel** package, so these should be installed first. If still there is an error, try to download the .tar or .zip (for Windows binaries) and install offline. These two files can be found at the CRAN website at https://cran.r-project.org/package=OmicsPLS. Also feel free to send an email with the error message you are receiving.

The OmicsPLS package is loaded by running `library(OmicsPLS)`. A message might be printed indicating that the `loadings` object is masked from `package::stats`. This basically means that whenever you type `loadings` (which is generic), you'll get the `loadings.o2m` variant. This is not a problem usually.

Background

The O2PLS method

The O2PLS method is proposed in (Trygg and Wold 2003):

$$X = TW^\top + T_\perp W_\perp^\top + E$$
$$\underbrace{Y}_{Data} = \underbrace{UC^\top}_{Joint} + \underbrace{U_\perp C_\perp^\top}_{Specific} + \underbrace{F}_{Noise}$$

It decomposes the variation of two datasets into three parts:

- A Joint part: $TW^\top$ for X and $UC^\top$ for Y,
- A Systematic/Specific/Orthogonal part: $T_\perp W_\perp^\top$ for X and $U_\perp C_\perp^\top$ for Y,
- A noise part: E for X and F for Y.

The number of columns in T, U, W and C are denoted by as n and are referred to as the number of joint components. The number of columns in $T_\perp$ and $W_\perp$ are denoted by as n_X and are referred to as the number of X-specific components. Analoguously for Y we use n_Y to denote the number of Y-specific components. The relation between T and U defines the relationship between X and Y: $U = TB_T + H_{UT}$ or $T = UB_U + H_{TU}$. Although this relationship seems asymmetric, the estimates are symmetric in X and Y. Ideally the number of components (n, n_X, n_Y) are known beforehand. If not the number of components can be selected with a data-driven method, for example Cross-Validation.

Cross-Validation

In cross-validation (CV) one minimizes a certain measure of error over some parameters that should be known a priori. In our case we have three parameters to determine a priori: (n, n_X, n_Y). A popular measure is the prediction error $||\hat{Y} - Y||$, where $\hat{Y}$ is a prediction of Y. However the O2PLS method is symmetric in X and Y, so we minimize the sum of the prediction errors: $||\hat{X} - X|| + ||\hat{Y} - Y||$. The idea is to fit O2PLS to our data X and Y and compute the prediction errors for a grid of values for n, n_X and n_Y. Here n should be a positive integer, and n_X and n_Y should be non-negative. The 'best' integers are then the minimizers of the prediction error.

Alternative cross-validation approach

We proposed an alternative way for choosing the number of components (Bouhaddani et al. 2016). First we construct a grid of values for n. For each n in this grid we consider the R^2 (coefficient of determination) between T and U for different n_X and n_Y. If T and U are contaminated with data-specific variation R^2 will be lower, as data-specific variation does not have predictive power. If too many specific components are removed R^2 will also be lower as also joint predictive variation is removed. The maximum R^2 is somewhere in between, yielding maximizers n_X and n_Y. With these two integers we compute the prediction error for our n that we have kept fixed. We repeat this process for each n on the one-dimensional grid and get our maximizers. This can provide a speed-up and often yields similar values for (n, n_X, n_Y).

Main functions

Brief overview

The functions in OmicsPLS can be organized as follows

- Cross-validation
- Fitting
- Summarizing & visualizing

For determining the number of components needed two Cross-Validation (CV) approaches are implemented: a standard approach and a faster alternative approach (see `?crossval_o2m` and `?crossval_o2m_adjR2`). After determining the number of components, an O2PLS fit is obtained by running `o2m` (type `?o2m` for the help page). The results can be inspected mainly by `summary` for the explained variantions and `plot` for the loadings.

Cross-validating

Two approaches for cross-validation are implemented. The standard CV is called by the following command

```
crossval_o2m(X, Y, a, ax, ay, nr_folds, nr_cores = 1, stripped = TRUE,
    p_thresh = 3000, q_thresh = p_thresh, tol = 1e-10, max_iterations = 100)
```

The first six arguments are mandatory. As in the `o2m` function, `X` and `Y` represent the two data sets. Instead of single integers we now have vectors of integers `a`, `ax` and `ay` that represent the number of columns. The number of folds is specified by `nr_folds`. It is recommended that at least ten folds are used. Too few folds (but not less than two) result in unreliable estimates. More folds are better, but then the computational cost is increased. A useful input parameter is `nr_cores`, the number of cores used, allowing for parallel computation on all platforms supported by the `parallel` package (Windows, Linux, OSM). The remaining arguments are directly passed on to `o2m`. There is no reason to set `stripped=FALSE` as this will only slow down the calculations.

The second CV approach is implemented in the function `crossval_o2m_adjR2`.

```
crossval_o2m_adjR2(X, Y, a, ax, ay, nr_folds, nr_cores = 1, stripped = TRUE,
    p_thresh = 3000, q_thresh = p_thresh, tol = 1e-10, max_iterations = 100)
```

It has exactly the same arguments as `crossval_o2m`. For this approach two folds were often enough to provide good values for `n`, `nx` and `ny`.

Fitting

The fitting function is `o2m`. It has five mandatory input parameters and more optional parameters. The full syntax is given by

```
o2m(X, Y, n, nx, ny, stripped = FALSE, p_thresh = 3000,
    q_thresh = p_thresh, tol = 1e-10, max_iterations = 100)
```

The matrices `X` and `Y` are the data, with rows as samples and columns as variables. The variables may be different, but each row must correspond to the same sample. The integers `n`, `nx` and `ny` are the number of components. Note that they must be non-negative, moreover `n` must be positive. The logical `stripped` indicates whether a stripped version of `o2m` should be used. The stripped version omits calculation and storage of the residual matrices E and F, which are as large as X and Y. The output of generic functions, e.g. `print`, `plot`, `summary`, remains the same. The integers `p_thresh` resp `q_thresh` are the minimum number of `X` resp `Y` variables for which `o2m` uses a memory-efficient NIPALS algorithm for high-dimensional data. By default `o2m` switches if both `X` and `Y` have 3000 columns. Note that the NIPALS approach is somewhat slower if one of the matrices is not high-dimensional (i.e. not many columns). The NIPALS approach is iterative, and `tol` (norm of the difference in loading values between two iterations) and `max_iterations` (maximum number of iterations) control termination of the algorithm. For many data sets it is sufficient to only specify the five mandatory arguments.

High dimensional fitting

In the `o2m` function the calculations of the joint components are based on the SVD of the cross-product $X^\top Y$. This can contain many elements if both matrices have many columns. For example when $p = q = 10000$ the number of elements in $X^\top Y$ is $pq = 10^8$ In these scenarios fitting the O2PLS method with SVD can be computationally not feasible. The `o2m` function can deal with data sets with many columns, by switching to the NIPALS algorithm (Wold 1973) for calculating the joint components. The NIPALS algorithm avoids the construction and storage of the covariance matrix $X^\top Y$, moreover the NIPALS-based joint components are equal to the SVD-based PLS components if the number of iterations are large enough (up to sign). In the case that p or q is not too large, the NIPALS approach is somewhat slower than the SVD approach.

Summarizing

To summarize the fitted variation different values can be reported by running the `summary` function on the object fitted with `o2m`.

```
summary(object, digits = 3, ...)
```

The `object` contains the `o2m` fit, while `digits` controls the amount of digits are printed. Among others, the following is printed.

- The variation of X explained by the joint or specific part is calculated as $||T||^2/||X||^2$ and $||T_\perp||^2/||X||^2$. Substituting T by U and X by Y yields formulas for Y.
- The variation of Y predicted by X is given by $||TB_T||^2/||X||^2$. Often it is more interesting to look at the variation of U predicted by T: $||TB_T||^2/||U||^2$. If only one component is present, this ratio equals the squared correlation between T and U. Similarly we obtain summary measures for Y.

- For assessing the predictive/explanatory power of the joint part of a subset of the observed variables, we can use the squared loadings as weights, as they sum up to one. The explained variation by the joint part is $||TW_S^\top||^2/||X||^2$ and for the predictive variation relative to U we have $||TBW_S^\top||^2/||U||^2$ for a subset of indices $S \subset \{1, \ldots, p\}$. For Y similar formulas hold.

Visualizing

The OmicsPLS package provides a function for plotting the loadings in each component. It uses on the {ggplot2} package, but a basic plot is also available if {ggplot2} is not available. The full command for plotting loadings is

`plot(x, loading_name, i, j, use_ggplot2, label, ...)`

Here `x` is the only required object, namely the O2PLS fit. All other input parameters have a default value. The parameter `loading_name` represents which of the four parts (X-joint, Y-joint, X-specific or Y-specific) should be plotted and should be one of `"Xjoint"`, `"Yjoint"`, `"Xorth"` or `"Yorth"`. The strings may be abbreviated to e.g. `"Xj"` (instead of `"Xjoint"`) as long as there is no ambiguity. The positive integers `i` and `j` denote which components to plot against each other. For plotting component i against its index, `j` can also be left unspecified. The `label` parameter can be one of two, either the index number if `label = "number"` or the variable names (if present in the data) if `label = "colnames"`. Also here the strings may be abbreviated to `"n"` and `"c"` respectively. Further arguments denoted by `...` will be processed by the plot function of {ggplot2}. Typically parameters like `col` (label color), `size` (label size), `alpha` (label transparancy) and/or `angle` (label angle) can be supplied here. The documentation of {ggplot2} contains much more information on this subject.

Real data example

We illustrate the OmicsPLS package with transcriptomic and metabolomic measurements from a Finnish population cohort, as part of the DILGOM study. The transcriptomic measurements can be found at ArrayExpress (http://www.ebi.ac.uk/arrayexpress/) under accession number `E-TABM-1036` (`E-TABM-1036.processed.1.zip`). The metabolite measurements are attached as supplemental material at (Inouye et al. 2010) (`msb201093-sup-0002.zip`).

Load the data

Now we download the data and prepare it in the right format (samples as rows and genes as columns) and give the rows and columns the right names. Note that this code chunk automatically downloads and loads the transcriptomic data into memory.

```
set.seed(31*12*2016)
if(!("test.tab" %in% list.files())){
  ## If you didn't download the expression data,
  ## this code will download it to the current directory (getwd())
  temp <- tempfile()
  download.file(
    "http://www.ebi.ac.uk/arrayexpress/files/E-TABM-1036/E-TABM-1036.processed.1.zip",
    temp)
  rna0 <- read.table(unzip(temp, "test.tab"), sep='\t')
  unlink(temp); rm(temp)
} else {
## Or if you've downloaded test.tab already we simply load it
  rna0 <- read.table("test.tab", sep='\t')
```

```
}
rna1 <- t(rna0[-(1:2),-1])
rna2 <- matrix(as.numeric(rna1), nrow = nrow(rna1))
dimnames(rna2) <- list(as.character(unlist(rna0[1,-1])),unlist(rna0[-(1:2),1]))
rna2 <- rna2[order(row.names(rna2)), ] # Order rows according to the participant ID
```

We define a function to pick only the top `100*prop` percent of the genes that have highest expression level, intersected with the top `100*prop` percent with highest Inter Quantile Range (see González et al. 2009). We apply it to our gene expression data, with `prop=0.75`.

```
filter_rna <- function(rna=rna, prop = 0.75){
  #calculate the maximum of gene expression per each gene and take the top
  maxGE <- apply(rna, 2, max)
  propGEmax <- quantile(maxGE, prop)
  #take the IQR of each gene and take the top genes
  IQRGE <- apply(rna, 2, IQR, na.rm=TRUE)
  propGEIQR <- quantile(IQRGE, prop)
  #selected genes/probes are the intersection of the two previous sets
  filter2 <- (intersect(which(maxGE> propGEmax), which(IQRGE> propGEIQR)))
  return(filter2)
}
rna3 <- rna2[,filter_rna(rna2)]
rm(rna0)
rm(rna1)
```

We also download and load the metabolite data and process it to have samples as rows and set the columns names.

```
if(!("metabonomic_data.txt" %in% list.files())){
  temp <- tempfile()
  download.file(
    "http://msb.embopress.org/content/msb/6/1/441/DC3/embed/inline-supplementary-material-3.zip",
    temp)
  metab0 <- read.table(unzip(temp, "metabonomic_data.txt"), header = T)
  unlink(temp); rm(temp)
} else {
  ## Or if you've downloaded metabonomic_data.txt already run the next line
  metab0 <- read.table("metabonomic_data.txt", header=T)
}
metab1 <- t(metab0[,-1])
colnames(metab1) <- metab0$Metabolite
```

Missing data imputation

Packages needed

- `install.packages("VIM")`
- `install.packages("missForest")`

Note that we have missingness in the metabolite data. The functions in OmicsPLS currently do not support missing data, as this is a delicate matter. Some diagostics on the missingness in the metabolite data can be obtained. Firstly we plot a histogram of the missing data. We need the `VIM` package for this.

```
VIM::aggr(metab1, col=c('navyblue','red'), numbers=TRUE, sortVars=FALSE,
          labels=names(data), cex.axis=.7, gap=3,
```

```
         ylab=c("Histogram of missing data","Pattern"))
```

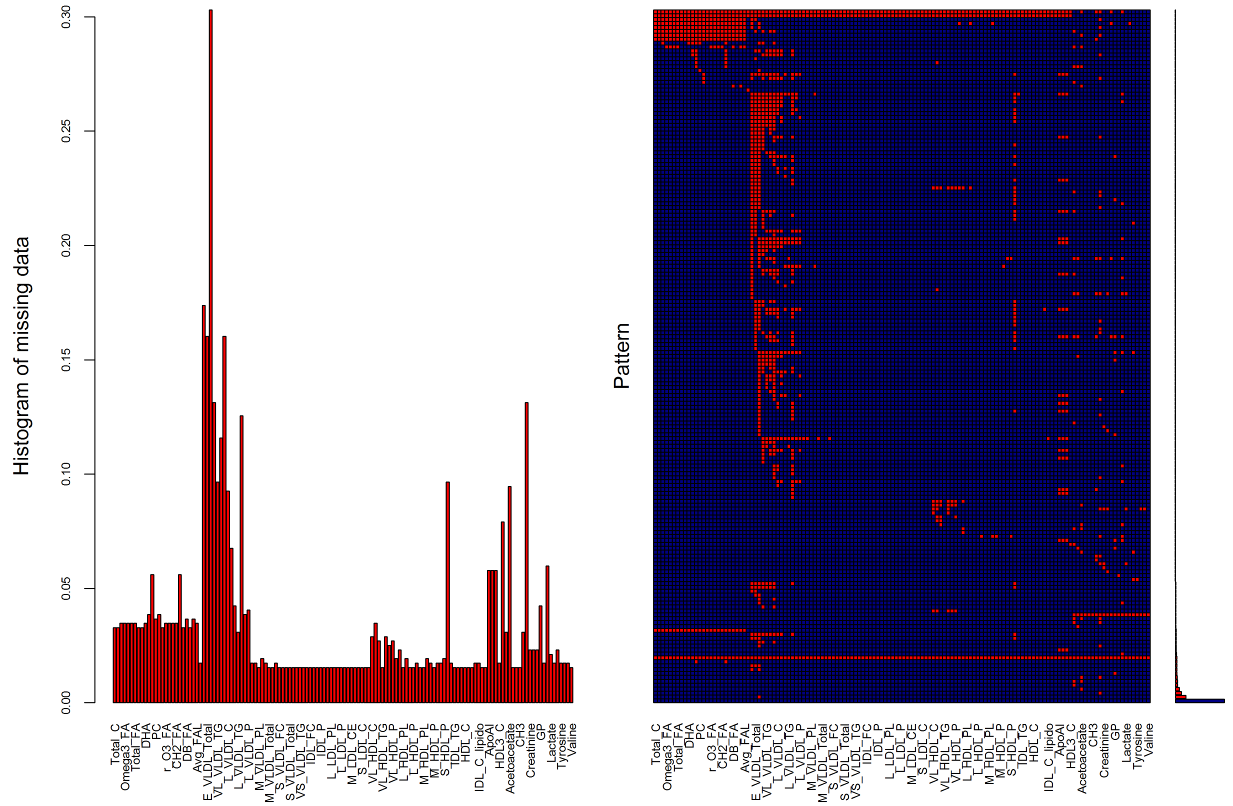

We remove participants with 100% missing metabolite measurements, i.e. missing rows.

```
NAs_in_metab1 <- which(apply(metab1, 1, function(e) sum(is.na(e))/length(e))==1)
metab2 <- metab1[-NAs_in_metab1,]
rna4 <- rna3[-NAs_in_metab1,]
```

Random Forests can be used to impute missing metabolites. We use the `missForest` package to do this. It takes some time, about 8 minutes on a modest i5 laptop, as can be seen from the output.

```
metab2.imp <- missForest::missForest(metab2, verbose = T)
```

```
##   missForest iteration 1 in progress...done!
##     estimated error(s): 0.4234906
##     difference(s): 0.02714137
##     time: 59.59 seconds
##
##   missForest iteration 2 in progress...done!
##     estimated error(s): 0.4189017
##     difference(s): 0.0005727861
##     time: 57.07 seconds
##
##   missForest iteration 3 in progress...done!
##     estimated error(s): 0.4185268
##     difference(s): 0.0002720186
##     time: 56.99 seconds
##
```

```
##   missForest iteration 4 in progress...done!
##     estimated error(s): 0.4203561
##     difference(s): 0.0002325965
##     time: 56.94 seconds
##
##   missForest iteration 5 in progress...done!
##     estimated error(s): 0.4195952
##     difference(s): 0.0002338311
##     time: 57.12 seconds
```

```
metab <- scale(metab2.imp$ximp, scale=F)
rna <- scale(rna4, scale = F)
```

In the last two lines, we took one imputed instance of the metabolite data and centered the columns of the RNA and metabolite data to have zero mean. We denote them by `rna` (transcripts) and `metab` (metabolites).

Inspect the data: descriptives

Packages needed

- `install.packages("gplots")`

A heatmap of metabolites, before and after imputation is plotted.

```
gplots::heatmap.2(cor(metab1,use = 'pair'), dendrogram='none', Rowv=F, Colv=F,trace='n',
                  breaks=seq(-1,1,length.out = 25), col=gplots::bluered)
gplots::heatmap.2(cor(metab,use = 'pair'), dendrogram='none', Rowv=F, Colv=F,trace='n',
                  breaks=seq(-1,1,length.out = 25), col=gplots::bluered)
```

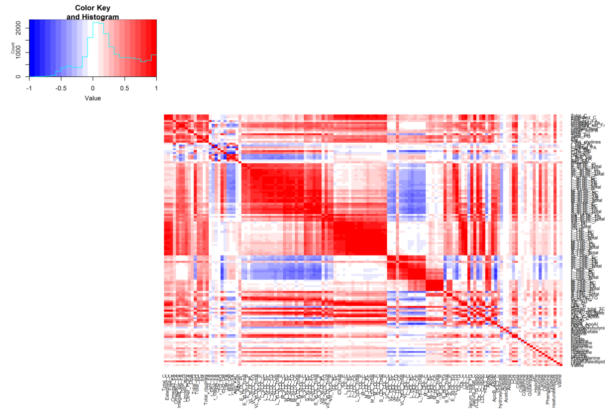

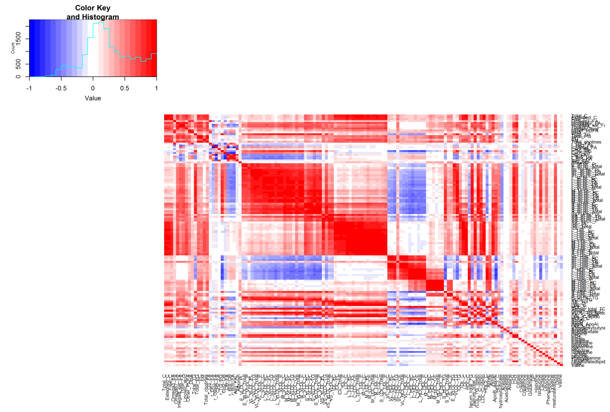

They are almost the same, indicating that the correlation structure within metabolites hasn't changed much.

To get an idea of the latent structure of the data we look at the eigenvalues of the covariance matrix of `rna` and `metab`.

```
## Eigenvalues within RNA
svd(rna, 0, 0)$d[1:6]^2 / sum(rna^2)
```

```
## [1] 0.19568455 0.12670559 0.09534211 0.05334638 0.03931151 0.03294359
```

```
## Eigenvalues within Metab
svd(metab, 0, 0)$d[1:6]^2 / sum(metab^2)
```

```
## [1] 0.37544553 0.21076846 0.10669151 0.04796332 0.03171004 0.02697083
```

```
## Singular values between RNA and Metab
svd(crossprod(rna,metab),0,0)$d[1:6]
```

```
## [1] 7109.291 3885.623 2453.775 2364.294 1990.450 1226.889
```

The first two commands calculate relative variances explained by each principal component. Strong latent structure is indicated by a sharp decline of the relative variances at the first few components. The last command calculates the singular values of the covariance between the two data sets. Also here, a strong decline in magnitude indicates strong latent structure in the covariance between the datasets.

Boxplots provide a good summary to compare the distribution of the variables relative to each other. Properties such as comparable means, variances and symmetry are often good to have. To reduce the number of boxplots we filter the transcriptomic data to include genes with 95% highest expression and IQR.

```r
boxplot(rna[,filter_rna(rna, .95)])
boxplot(metab)
```

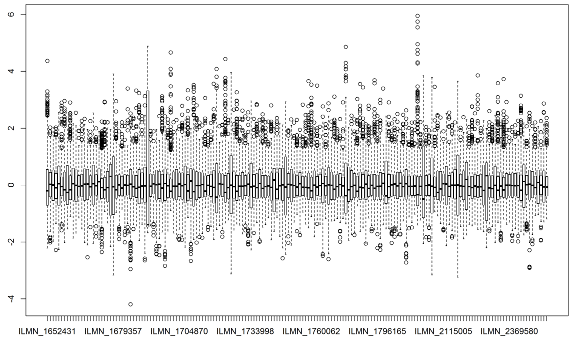

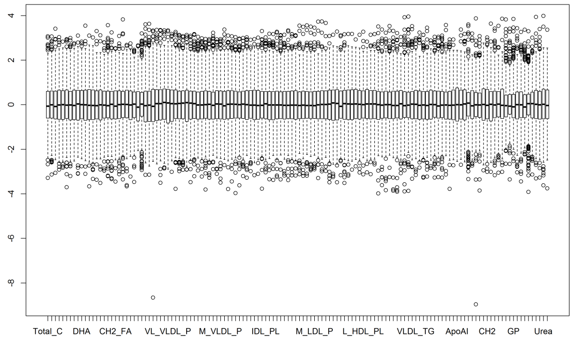

The distributions are quite symmetric and the scale is comparable across variables in each data set.

Analysis with the OmicsPLS package

Cross-validation

We load the OmicsPLS package and set a seed for the cross-validation. The strategy is to define a relatively large grid to search on and apply the faster alternative Cross-Validation (Cv) approach to find a solution. Then on a smaller grid containing these best integers we do a full CV to determine the best choice for the number of components. The objective function to minimize is the sum of the two prediction errors $||X - \hat{X}||$ and $||Y - \hat{Y}||$.

```r
library(OmicsPLS)
```

```
##
## Attaching package: 'OmicsPLS'

## The following object is masked from 'package:stats':
##
##     loadings
```

```r
set.seed(1+1+2016)
CV1 <- crossval_o2m_adjR2(rna, metab, 1:3, c(0,1,5,10), c(0,1,5,10),
                          nr_folds = 2, nr_cores = 4)
```

```
## minimum is at n = 2

## Elapsed time: 66.87 sec
```

```r
CV2 <- crossval_o2m(rna, metab, 1:2, 0:2, 9:11,
                    nr_folds = 10, nr_cores = 4)
CV1
```

```
##        MSE n nx ny
## 1 1.297685 1  1 10
## 2 1.293595 2  1 10
## 3 1.323277 3  1 10
```

```r
CV2
```

```
## *******************
## Elapsed time: 187.9 sec
## *******
## Minimal 10-CV error is at ax=1 ay=10 a=2
## *******
## Minimum is 1.281668
## *******************
```

Following the advice of the last CV output, we select two joint, one transcript-specific and ten metabolite-specific components. We fit the O2PLS model with default values as follows.

```r
library(OmicsPLS)
```

```
##
## Attaching package: 'OmicsPLS'

## The following object is masked from 'package:stats':
##
##     loadings
```

```r
fit = o2m(rna, metab, 2, 1, 10)
fit
```

```
## O2PLS fit
## with 2 joint components
## and  1 orthogonal components in X
## and  10 orthogonal components in Y
## Elapsed time: 1.92 sec
```

The total runtime of the fit was about 3 seconds. Note that univariate correlation tests would require almost one million tests to be performed, and does not take into account correlation between metabolites and genes. Also multivariate linear regression cannot deal with the large amount of variables.

A summary of the results is obtained via

```r
summary(fit)
```

```
##
## *** Summary of the O2PLS fit ***
##
## -  Call: o2m(X = rna, Y = metab, n = 2, nx = 1, ny = 10)
##
## -  Modeled variation
## -- Total variation:
## in X: 332035.7
## in Y: 68381.71
##
## -- Joint, Orthogonal and Noise as proportions:
```

```
##
##            data X data Y
## Joint       0.124  0.410
## Orthogonal  0.026  0.062
## Noise       0.850  0.528
##
## -- Predictable variation in Y-joint part by X-joint part:
## Variation in T*B_T relative to U: 0.151
## -- Predictable variation in X-joint part by Y-joint part:
## Variation in U*B_U relative to T: 0.116
##
## -- Variances per component:
##
##           Comp 1    Comp 2
## X joint 25039.63 16203.728
## Y joint 18456.89  9555.336
##
##          Comp 1
## X Orth 56637.85
##
##          Comp 1   Comp 2   Comp 3   Comp 4   Comp 5   Comp 6  Comp 7
## Y Orth 6715.068 3315.059 2055.242 1199.923 1091.131 1103.341 838.014
##         Comp 8  Comp 9 Comp 10
## Y Orth 950.716 570.621 850.223
##
##
## -  Coefficient in 'U = T B_T + H_U' model:
## -- Diagonal elements of B_T =
##  0.401 0.339
```

The joint, orthogonal and noise variations are shown as proportions. The two joint components explains about 12% of the transcriptomic variation and 41% of the metabolite variation, these proportions are 17% and 24% for the orthogonal part. We also observe that *relative to the variation in U*, the variation predicted by T (or equivalently X, transcripts) is 11.6%. Looking relative to the variation in Y (metabolites), the variation predicted by T (or equivalently X) is $0.116 * 0.41$. Similar calculations can be performed for the Y part.

Plotting

Packages needed

- `install.packages("magrittr")`
- `install.packages("ggplot2")`
- `install.packages("gridExtra")`
- `install.packages("stringr")`
- `install.packages("gplots")`
- `install.packages("reshape2")`

We want to see which (groups of) metabolites and transcripts tend to correlate with each other. To do this we plot the loadings. The individual loading values per component indicate the relative importance of each variable to the corresponding component. We plot the two joint loadings against each other to see which metabolites are most important for each component. To do this we need three packages for convenience: `magrittr` for the piping operator, `ggplot2` for plotting and `gridExtra` to put multiple ggplots in one figure. Also `stringr` will be needed to extract substrings of column names. The `reshape2` package is needed for reshaping data sets from wide format to long format.

```r
library(magrittr)
library(ggplot2)
library(gridExtra)
library(illuminaHumanv3.db)
```

```
## Warning in rsqlite_fetch(res@ptr, n = n): Don't need to call dbFetch() for
## statements, only for queries
```

```r
# Color names
LLmodule <- c("ILMN_1690209",'ILMN_1766551', 'ILMN_1749131', 'ILMN_1688423',
              'ILMN_2102670', 'ILMN_1792323', 'ILMN_1899034', 'ILMN_1806721',
              'ILMN_1695530', 'ILMN_1726114', 'ILMN_1751625', 'ILMN_1726114',
              'ILMN_1753648', 'ILMN_1779043')
LLnr <- which(colnames(rna) %in% LLmodule)
rna_genenames <- select(illuminaHumanv3.db,
                        keys = colnames(rna)[LLnr],
                        keytype = "PROBEID", columns = "SYMBOL")[,2]
```

```
## Warning in rsqlite_fetch(res@ptr, n = n): Don't need to call dbFetch() for
## statements, only for queries

## Warning in rsqlite_fetch(res@ptr, n = n): Don't need to call dbFetch() for
## statements, only for queries
```

```r
name_col <- 1 + sapply( #First sapply loops over column names
  X = colnames(metab),
  FUN = function(arg){
    crossprod(
      c(1, 1, 3, 4, 5), # Weights to be used as categories
      sapply(c("VLDL", "LDL", "IDL", "HDL","FA"), # metabolite classes
             function(arg2){grepl(arg2, arg)} # compare class of metabolites
      )
    )
    }
  )
name_col <- factor(name_col,
                   levels = c(3,2,4:6,1),
                   labels = c("VLDL", "LDL", "IDL", "HDL","FA","Other"))

# alpmetab <- loadings(fit, "Yjoint", 1:2) %>%  # Retreive loadings
#   abs %>% # Absolute loading values for positive weights
#   rowSums %>% # Sum over the components
#   sqrt + (name_col!="Other") # Take square root

######### Plot loadings with OmicsPLS plot method ###
p_metab <- plot(fit, loading_name="Yj", i=1, j=2, label="c", # Plot the loadings
             alpha=0) + # set points to be 100% transparant
##################### Add all layers ###
  theme_bw() +
  coord_fixed(ratio = 1, xlim=c(-.2,.2),ylim=c(-.2,.2)) +
  geom_point( # Set color and size
    aes(col=name_col, size = I(1+(name_col%in%c("VLDL","HDL"))),
        shape = name_col),show.legend = T) +
  theme(legend.position="right") +
  scale_color_discrete(name="Metabolite\nGroup",
```

```
                         labels=c("VLDL", "LDL", "IDL", "HDL","FA","Other")) +
  guides(size=F) + scale_shape_discrete(name="Metabolite\nGroup",
                                  labels=c("VLDL", "LDL", "IDL", "HDL","FA","Other")) +
  scale_shape_manual(name="Metabolite\nGroup", values=c(15,3,4,17,5,6)) +
  labs(title = "Metabolite joint loadings",
       x = "First Joint Loadings", y = "Second Joint Loadings") +
  theme(plot.title = element_text(face='bold'),
        legend.title=element_text(face='bold')) +
  geom_hline(yintercept = 0) + geom_vline(xintercept = 0)

alprna <- loadings(fit, "Xjoint", 1:2) %>% raise_to_power(2) %>% rowSums
alprna[-(order(alprna,decreasing=T)[1:10])] = 0
alprna <- sign(alprna)
toprna <- which(alprna>0)
names_rna <- mapIds(illuminaHumanv3.db,
       keys = colnames(rna)[toprna],
       keytype = "PROBEID",
       column = "SYMBOL",
       multiVals = 'first')
```

```
## Warning in rsqlite_fetch(res@ptr, n = n): Don't need to call dbFetch() for
## statements, only for queries

## Warning in rsqlite_fetch(res@ptr, n = n): Don't need to call dbFetch() for
## statements, only for queries
```

```
names_rna[which(is.na(names_rna))] <- "?"
######### Plot loadings with OmicsPLS plot method ###
p_rna <- ggplot(data.frame(x = fit$W.[, 1], y = fit$W.[, 2]),
                aes(x = x, y = y),
                alpha = alprna,
                aes(label = NA)) +
    ##################### Add all layers ###
  theme_bw() +
  coord_fixed(.8, c(-.15,.15),c(-.15,.15)) +
  geom_point(alpha = 0.5, col = 'grey') +
  geom_point(data = data.frame(x=fit$W.[LLnr,1],y=fit$W.[LLnr,2]),
             shape = 2, col = 2, size = 2) +
  geom_text(data = data.frame(x=fit$W.[toprna,1],y=fit$W.[toprna,2]),
            hjust = rep(c(1, 0), length.out = length(toprna)),
            aes(label = names_rna)) +
  labs(title = "Transcript joint loadings",
       x = "First Joint Loadings", y = "Second Joint Loadings") +
  theme(plot.title = element_text(face='bold')) +
  geom_hline(yintercept = 0) + geom_vline(xintercept = 0)

## Finally plot both plots in one figure.
grid.arrange(p_metab, p_rna, ncol=2)
```

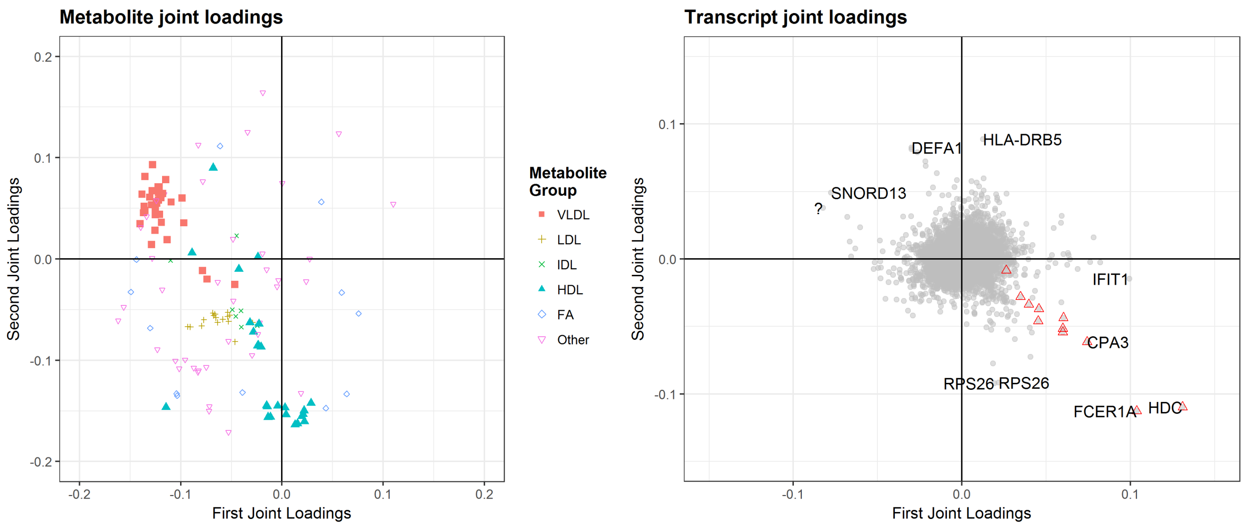

The genes with highest absolute loading values are most related with the metabolites having highest absolute loading values on the respective axes. It can be seen that especially VLDL metabolites cluster together in both axes, indicating that are correlated within both joint components. Moreover in the second component they tend to be negatively correlated to HDL metabolites. The VLDL metabolites are most correlated with expression of the *HDC* gene in the first component. In the second component the VLDL and HDL metabolites are most correlated with expression of genes involved in defense response and inflammation (e.g. *FCER1A*, *HDC* and *DEFA1*).

CPU times

Packages needed

- `install.packages("microbenchmark")`

In OmicsPLS we added an alternative, memory-efficient, fitting algorithm (NIPALS) for high-dimensional data. This omits storing the whole covariance matrix of size p times q. In case p and q are large, say larger than 3000 both, storing this becomes a memory intensive operation. To see how long `o2m` takes to fit, we consider three scenarios. They are timed with the `microbenchmark` function.

```
set.seed(2016^2)
fake_X <- scale(matrix(rnorm(1e2*1e4),1e2)) # 100 x 10000 matrix
fake_Y <- scale(matrix(rnorm(1e2*1e2),1e2)) # 100 x 100 matrix
suppressMessages(
  scenario1 <- microbenchmark::microbenchmark(
    default=o2m(fake_X, fake_Y, 1, 1, 1),
    stripped=o2m(fake_X, fake_Y, 1, 1, 1, stripped=T),
    highD = o2m(fake_X, fake_Y, 1, 1, 1, stripped=T, p_thresh=1),
    times = 6, unit = 's',control=list(warmup=1))
```

```
)
scenario1
```

```
## Unit: seconds
##       expr       min       lq      mean    median        uq       max neval
##    default 0.8184800 0.818827 0.8357437 0.8279945 0.8531872 0.8679787     6
##   stripped 0.8115051 0.832465 0.8529599 0.8490492 0.8765673 0.8991234     6
##      highD 2.0267014 2.046781 2.1124864 2.0558910 2.2090854 2.2805691     6
##   cld
##    a
##    a
##     b
```

First two data sets are generated, having 100 rows. The first data set has 10000 columns, the second data set has 100 columns. The first row corresponds to an `o2m` fit with default settings. In the second row a stripped version of the algorithm is used, i.e. no noise matrices are calculated. For low-dimensional this does not matter much in CPU time. The last row corresponds to a fit using the NIPALS algorithm, which is only advantageous for high dimensional data. This version of `o2m` is somewhat slower.

```
fake_X <- scale(matrix(rnorm(1e2*2e3),1e2)) # 100 x 2000 matrix
fake_Y <- scale(matrix(rnorm(1e2*2e3),1e2)) # 100 x 2000 matrix
suppressMessages(
  scenario2 <- microbenchmark::microbenchmark(
    default=o2m(fake_X, fake_Y, 1, 1, 1),
    stripped=o2m(fake_X, fake_Y, 1, 1, 1, stripped=T),
    highD = o2m(fake_X, fake_Y, 1, 1, 1, stripped=T, p_thresh=1),
    times = 6, unit = 's',control=list(warmup=1))
)
scenario2
```

```
## Unit: seconds
##       expr       min        lq      mean   median        uq       max neval
##    default 39.463320 40.318531 40.459705 40.43632 40.680007 41.423723     6
##   stripped 40.334436 40.373511 41.026917 40.98918 41.706695 41.768499     6
##      highD  1.078915  1.094818  1.107499  1.11006  1.125557  1.125582     6
##   cld
##     b
##     b
##    a
```

Here 'medium-dimensional' data sets are generated, having 100 rows and 2000 columns. In this scenario the NIPALS approach outperforms the 'default' approach.

```
fake_X <- scale(matrix(rnorm(1e2*5e4),1e2)) # 100 x 50000 matrix
fake_Y <- scale(matrix(rnorm(1e2*5e4),1e2)) # 100 x 50000 matrix
o2m(fake_X, fake_Y, 1, 1, 1, stripped=T, p_thresh=1e6)
```

```
## Error: cannot allocate vector of size 18.6 Gb
```

```
o2m(fake_X, fake_Y, 1, 1, 1, stripped=T)
```

```
## Using high dimensional mode with tolerance 1e-10 and max iterations 100

## Power Method (comp 1) stopped after 100 iterations.

## Power Method (comp 2) stopped after 100 iterations.

## Power Method (comp 1) stopped after 100 iterations.
```

```
## O2PLS fit: Stripped
## with 1 joint components
## and  1 orthogonal components in X
## and  1 orthogonal components in Y
## Elapsed time: 15.07 sec
```

```r
rm(fake_X)
rm(fake_Y)
```

Here high-dimensional data sets are generated, having 100 rows and 50000 columns. Fitting O2PLS is perfectly possible with the NIPALS approach, but infeasible with the default approach.

References

Bouhaddani, S. el, J. Houwing-Duistermaat, P. Salo, M. Perola, G. Jongbloed, and H.-W. Uh. 2016. "Evaluation of O2PLS in Omics data integration." *BMC Bioinformatics* 17 Suppl 2 (2):11. https://doi.org/10.1186/s12859-015-0854-z.

González, I., S. Déjean, P. G. P. Martin, O. Gonçalves, P. Besse, and A. Baccini. 2009. "Highlighting Relationships Between Heterogeneous Biological Data Through Graphical Displays Based on Regularized Canonical Correlation Analysis." *Journal of Biological Systems* 17 (02):173–99. https://doi.org/10.1142/S0218339009002831.

Inouye, Michael, Johannes Kettunen, Pasi Soininen, Kaisa Silander, Samuli Ripatti, Linda S Kumpula, Eija Hämäläinen, et al. 2010. "Metabonomic, Transcriptomic, and Genomic Variation of a Population Cohort." *Molecular Systems Biology* 6 (1). John Wiley & Sons, Ltd. https://doi.org/10.1038/msb.2010.93.

Trygg, J., and S. Wold. 2003. "O2-Pls, a Two-Block (X-Y) Latent Variable Regression (Lvr) Method with an Integral Osc Filter." *Journal of Chemometrics* 17 (1). John Wiley & Sons, Ltd.:53–64. https://doi.org/10.1002/cem.775.

Wold, H. 1973. "Nonlinear Iterative Partial Least Squares (NIPALS) Modelling: Some Current Developments." In *Multivariate Analysis, III (Proc. Third Internat. Sympos., Wright State Univ., Dayton, Ohio, 1972)*, 383–407. New York: Academic Press.
